# Supplementary material for: Dynamical strengthening of covalent and non-covalent molecular interactions by nuclear quantum effects at finite temperature
Source: Nat Commun. 2021 Jan 19;12:442. doi: 10.1038/s41467-020-20212-1 (PMC7815839; doi:10.1038/s41467-020-20212-1)
Supplement: Supplementary file 1 — Supplementary Information [file 41467_2020_20212_MOESM1_ESM.pdf]

SUPPLEMENTARY INFORMATION: Dynamical Strengthening of Covalent and Non-Covalent Molecular Interactions by Nuclear Quantum Effects at Finite Temperature

Huziel E. Sauceda,[1, 2, 3, *] Valentin Vassilev-Galindo,[1] Stefan Chmiela,[2] Klaus-Robert Müller,[2, 4, 5, 6, †] and Alexandre Tkatchenko[1, ‡]

[1] *Department of Physics and Materials Science, University of Luxembourg, L-1511 Luxembourg, Luxembourg*
[2] *Machine Learning Group, Technische Universität Berlin, 10587 Berlin, Germany*
[3] *BASLEARN, BASF-TU joint Lab, Technische Universität Berlin, 10587 Berlin, Germany*
[4] *Department of Artificial Intelligence, Korea University, Anam-dong, Seongbuk-gu, Seoul 02841, Korea*
[5] *Max Planck Institute for Informatics, Stuhlsatzenhausweg, 66123 Saarbrücken, Germany*
[6] *Google Research, Brain team, Berlin, Germany*

(Dated: November 10, 2020)

SUPPLEMENTARY INFORMATION

Supplementary Note 1. Reference data generation

The coupled cluster (CC) reference data used for training the models was created by creating a representative subsampling [1] of the MD17 datasets [2] for the three molecules under study. For the toluene molecule, the same procedure was followed to generate the data at the Hartree-Fock/aug-cc-pVQZ level of theory and density functional theory (DFT:PBE0+MBD/*really_tight*).

The CC data was generated by single-point force and energy calculations using all-electron coupled cluster with single, double, and perturbative triple excitations (CCSD(T)/cc-pVDZ) for toluene. In the case of aspirin, CC calculations up to double exitations (CCSD) were done using Dunning's correlation-consistent basis set cc-pVDZ. All CC calculations were done using the interactive quantum chemistry programming environment Psi4 [3–5]. For paracetamol, we have used PBE0+MBD/*really_tight* given that it provides better energetic values relative to CCSD(T)/cc-pVDZ than CCSD/cc-pVDZ as shown in Table. I The DFT calculations were performed using the FHI-aims software [6]. All our calculations, including CC and DFT, were done using all electrons.

Supplementary Note 2. Numerically tabulated atom-centered orbitals

The Dunning's correlation-consistent basis set is a well established basis, then not much detail has to be included in this sections to describe their accuracy and capabilities. On the other hand, numerically tabulated atom-centered orbitals (NAOs), as implemented the FHIaims package, are less known. NAOs basis sets in the FHIaims package are constructed to be transferable and hierarchical basis sets to systematically reach basis set convergence limit (sub-meV-level accuracy on the total energy). The basis sets are constructed starting from a minimal basis set (minimal free-atom basis) and then systematically adding new energetically-favorable functions from a pool of basis functions (hydrogen-like, cation-like, and atom-like functions with a variable confinement potential). Then, the basis used in this work, named "*really_tight*" in the code, consists in, for example the hydrogen like functions for C[{minimal}+H(nl,z)]: {[He]+2s 2p}+H(4f,9.8). A more detailed description can be found in the original article by Blum *et al.* Comput. Phys. Commun. 180, 2175 (2009). [6] In general, the *really_tight* basis set computed energies are expected to be converged at a level of few meV, for example in the case of the molecular system $(H_2O)_2$, the error achieved with this basis set is ∼1 meV/atom, which for practical terms is converged with respect to the basis set size. Based on this and the extended analysis presented in the original publication, it is expected that molecular calculation using this atomic basis set are expected to give near converged results.

SUPPLEMENTARY TABLE I. Energies relative to the global minimum of the local minimum (local) and the transition state (TS). The energies are in kcal mol^{-1} computed with different levels of theory. The CCSD and CCSD(T) were calculated using the cc-pVDZ basis set and the DFT calculation used *really_tight* basis as implemented in FHIaims. [6]

Molecule	Level of theory		
paracetamol	CCSD	CCSD(T)	PBE0+MBD
Local	0.3836	0.3788	0.3696
TS	3.0640	3.4445	3.4475
TS - Local	2.6804	3.0657	3.0780

* sauceda@tu-berlin.de
† klaus-robert.mueller@tu-berlin.de
‡ alexandre.tkatchenko@uni.lu

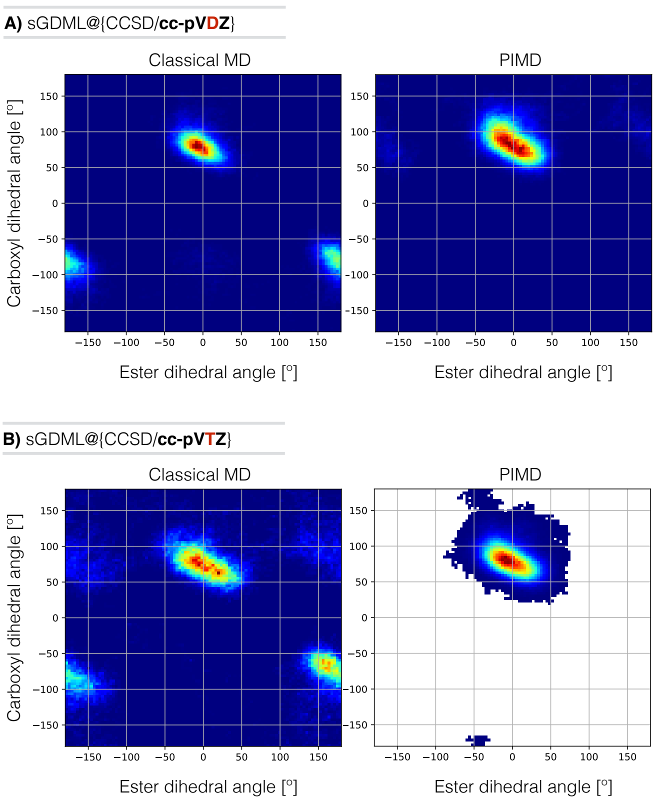

SUPPLEMENTARY FIGURE 1. Classical (MD) and path integral molecular dynamics (PIMD) simulations at room temperature of aspirin described by the sGDML@CCSD molecular force field using the basis set cc-pVDZ (A) and cc-pVTZ (B). The plots are projections of the dynamics to the two main degrees of freedom of aspirin: carboxyl and ester dihedral angles.

Supplementary Note 3. Calculation of $E_{n\to\pi^*}$

Natural Bond Orbital (NBO) second order perturbative energies $E_{n\to\pi^*}$ obtained from NBO 7.0 [7] calculations coupled with ORCA 4.1.2 [8, 9] at CCSD/cc-pVDZ level of theory were taken as the stabilization energies due to $n \to \pi^*$ interactions. Such interactions, which play an important role in molecular reactivity and conformation (for instance, the Bürgi-Dunitz trajectory [10] preferred during nucleophilic attacks at a carbonyl carbon), comprise delocalization of lone-pair electrons (n) of an electronegative atom into an empty π^*–antibonding orbital of an aromatic ring or a carbonyl group [11–13].

Supplementary Note 4. Statistical convergence with the basis set size

For small molecules considered in our work, the convergence of their thermodynamical and statistical properties do not require large basis sets. To show this, Supplementary Figure 1 presents the results of classical MD and PIMD simulations using a sGDML model for aspirin trained on CCSD/cc-pVDZ and CCSD/cc-pVTZ reference calculations, which display statisticaly the same behaviour. This means that intramolecular interactions in small molecules are already statistically well captured with cc-pVDZ basis set.

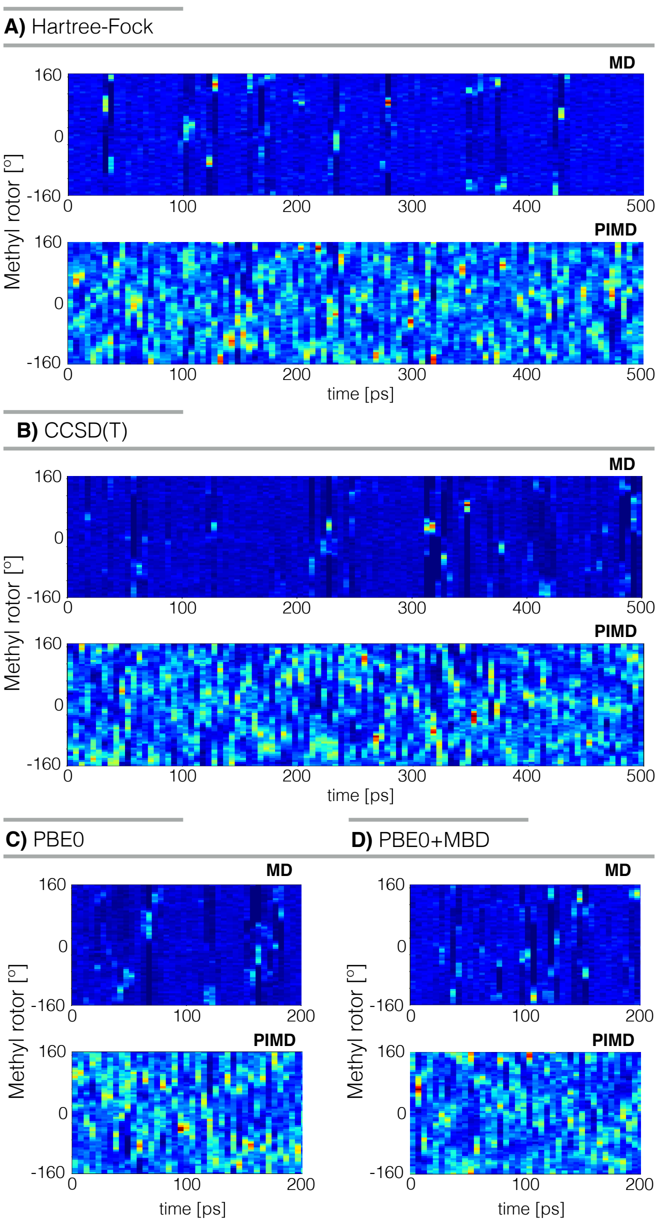

SUPPLEMENTARY FIGURE 2. Comparison of the time evolution of the methyl rotor dynamics in toluene using classical MD and PIMD. Four different levels of theory were used to describe the potential energy surface, A) Hartree-Fock (HF), B) coupled cluster with single, double, and perturbative triple excitations (CCSD(T)), and density functional theory using the C) PBE0 functional and D) PBE0+MBD. The size of the bins are 5 ps × 6°.

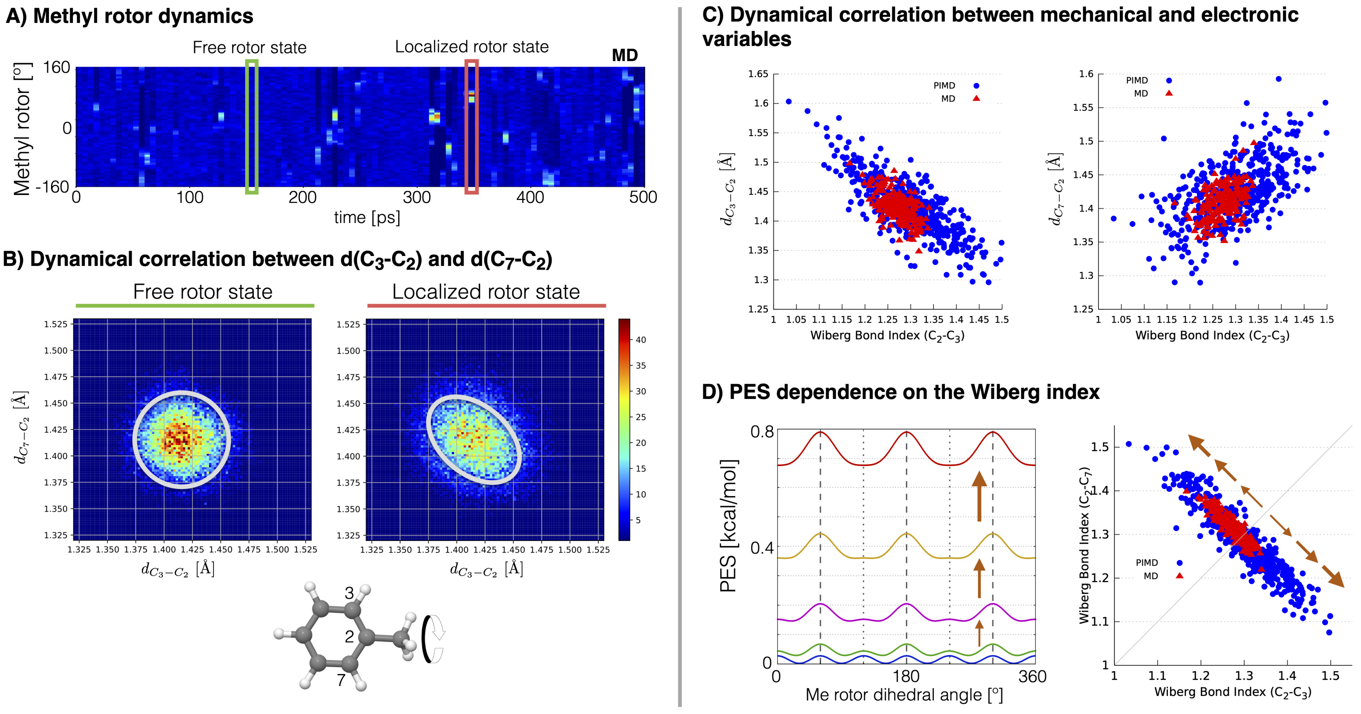

SUPPLEMENTARY FIGURE 3. A) Free (green) and localized (red) states in a classical MD simulation of the methyl rotor in the toluene molecule. The size of the bins are 5 ps × 6°. B) Correlation between the bond lengths $d_{C_3-C_2}$ and $d_{C_7-C_2}$ during the free and localized states of the methyl rotor. C) Dynamical correlation between the Wiberg's bond index for the bond $C_3 - C_2$ and bond length $d_{C_3-C_2}$ (left) and bond length $d_{C_7-C_2}$ (right). The classical MD (red) is contrasted with the PIMD (blue) simulations. The simulations were done at room temperature at the sGDML@CCSD(T) level of theory. D) Methyl rotor's PES for different fixed values of the C_2-C_3 distance (left) and its qualitative relationship with the Wiberg bond index (right).

Supplementary Note 5. Molecular dynamics settings

The molecular dynamics simulations were done using the i-PI code [14, 15] with the sGDML force field interface [16]. The integration time-step was set to 0.2 fs using the NVT ensemble and the total simulation time was 0.5 ns. All the simulations were performed at room temperature.

The path integral molecular dynamics (PIMD) simulations were performed using a baseline of 16 beads, value which in most cases gives converged thermodynamic properties of molecules at room temperature [17–19]. Such convergence was corroborated by running shorter PIMD trajectories (100 ps instead of 0.5 or 1 ns) using 32 beads. In the particular case of the methyl rotor dynamics in toluene, given the unexpected localization, the convergence of the results was validated running simulations using up to 64 beads.

Supplementary Note 6. Methyl rotor analysis: Different levels of theory

As mentioned in the main text, the rotor localization does not depend on the level of theory used to describe the PES of the toluene rotor localization. Supplementary Figure 2 shows direct evidence of this statement. The most evident case is that Hartree-Fock (HF/aug-cc-pVQZ) displays the same trends as CCSD(T)/cc-pVDZ even though their rotational barriers are 0.0008 kcal mol^{-1} and 0.028 kcal mol^{-1}, respectively. The same results are obtained using DFT with PBE0 and PBE0+MBD. The many body dispersion (MBD) [20, 21] treatment of the van der Waals interaction [22, 23] increases the PBE's rotational barrier given its tight relationship to the correlation energy. Despite the differences in the four levels of theory used for the simulation, the methyl rotor hindering was proven to be level-of-theory agnostic.

All the PIMD simulations in Supplementary Figure 2 were done using 16 beads, but they were validated with shorter simulations with upto 64 beads.

Supplementary Note 7. Origin of the rotor energetic barrier

From the electronic structure point of view, previous studies suggested that [24–27] the Me rotational energy barrier E_{MeRot} originates from the difference of the π-bond order between the two ring bonds C_2-C_3 and

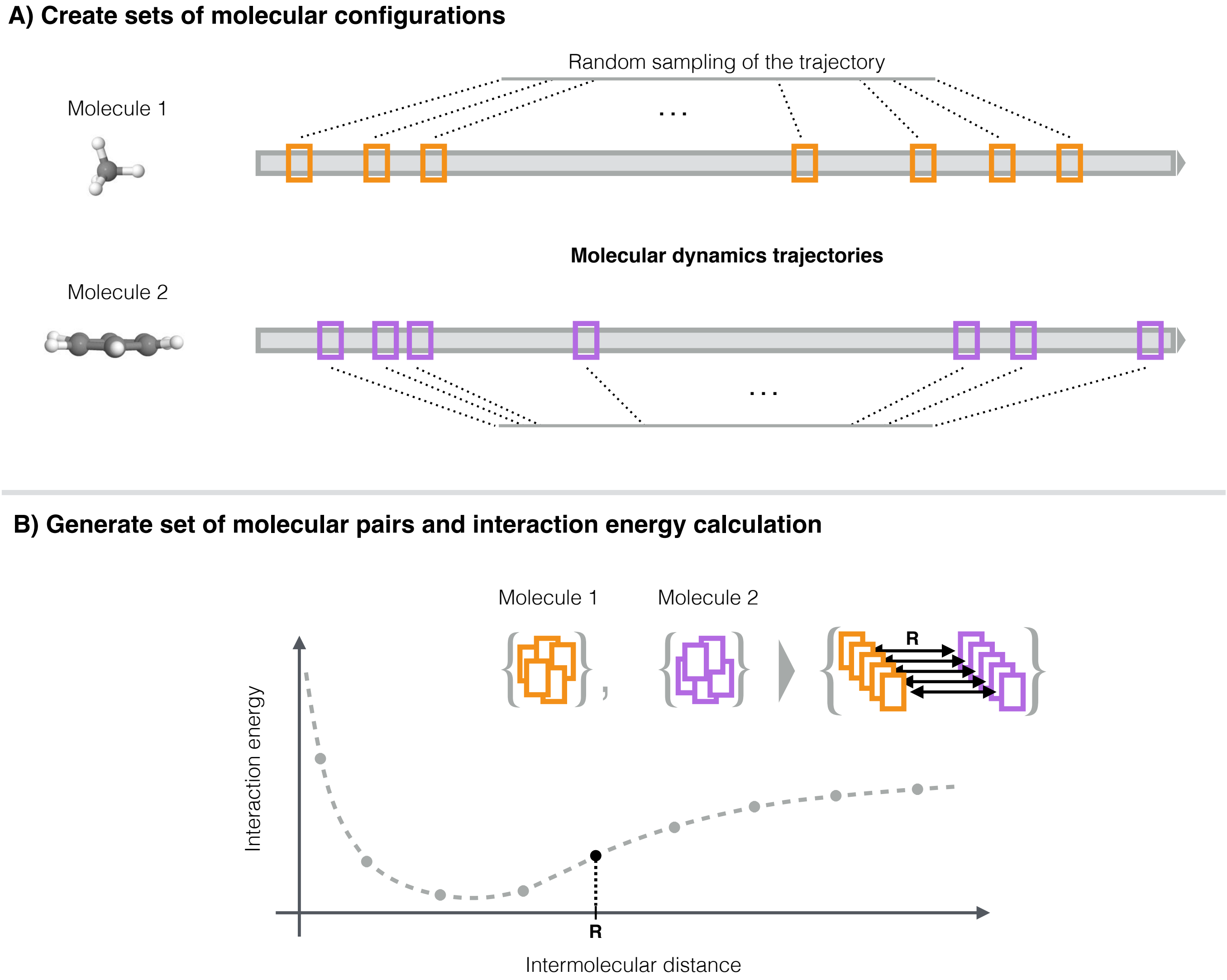

SUPPLEMENTARY FIGURE 4. Diagram for creating the noncovalent interactions dataset. A) From a pair of individual molecular dynamics trajectories performed under the same conditions (e.g. temperature, thermostat, and level of theory) for molecules 1 and 2, each trajectory is randomly sampled to collect a dataset of snapshots of molecular configurations (rectangles in orange and purple). Each one of the molecular datasets has N molecular configurations. B) From the two datasets, one for each molecule, a dataset of randomly selected molecular pairs is assembled separated at a distance R. Then, the intermolecular interaction energy is computed for the N molecular pairs, averaged and plotted as a function of R. The same procedure is repeated for several values of R.

C_2–C_7 near to the Me group (Supplementary Figure 3-B) [25]. To corroborate this and to assess its behaviour at finite temperatures, we have monitored the Wiberg Bond Indices (WBI) of these bonds computed with NBO 7.0 [7] during a Me localized state (Supplementary Figure 3-A) for classical MD and PIMD. The correlation $\mathrm{WBI}_{C_2-C_3} \sim -\mathrm{WBI}_{C_2-C_7}$ in this plot agrees with the linear correlations between their corresponding bond lengths (i.e. $d_{C_2-C_3} \sim -d_{C_2-C_3}$) in a localized Me rotor state, giving the direct relationship $\mathrm{WBI}_{C_2-C_3} \sim -d_{C_2-C_3} \sim +d_{C_2-C_7}$ (see Supplementary Figure 3-C). In this context, the electron gain/depletion (bond length decrease/increase) drastically changes the energy landscape. Computing $d_{C_2-C_3}$-restricted Me rotor PES (Supplementary Figure 3-D) gives a lower bound estimate for the increase of the E_{MeRot}, which can be up to ∼400% larger than the reference (∼0.028 → 0.120 kcal mol^{-1}). From these results we can conclude that the NQE+thermal fluctuations boost the energy barriers generating the Me hindered dynamics.

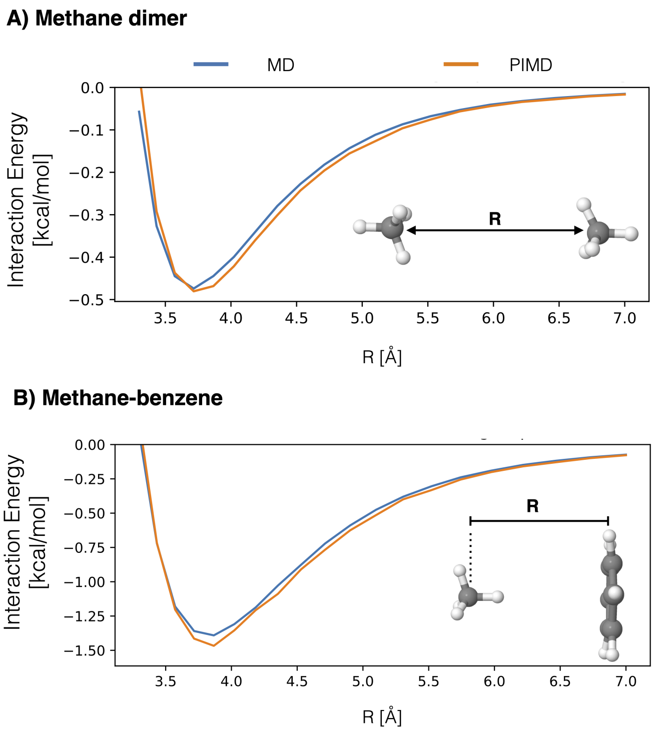

SUPPLEMENTARY FIGURE 5. Strengthening of noncovalent interactions in A) methane dimer and B) methane–benzene system by NQE at room temperature.

Supplementary Note 8. Noncovalent interactions data preparation

In order to analyse the noncovalent interaction energy in a systematic way, a set of molecular pairs separated by regular intermolecular distance (R) intervals has to be created. An approximation to such curve can be obtained by sampling MD trajectories of individual molecules and then create molecular pairs from them. Supplementary Figure 4 shows the followed procedure. First, from a MD trajectory (classical or quantum) for molecule 1 simulated at a given temperature and level of theory, a set of N randomly sampled molecular configurations is created. This is repeated for molecule 2. The two molecules can be different or the same. This is illustrated in Supplementary Figure 4-A. From the two datasets, a new dataset of randomly selected molecular pairs is assembled enforcing a given intermolecular separation R and relative molecular orientation depending of which molecular arrangement wants to be analyzed, for example parallel benzene dimer, and separated at a distance R. Once the dataset of molecular pairs was created, the intermolecular interaction energy for each of the N molecular pairs is computed. Here, we have used symmetry-adapted perturbation theory (SAPT)/aug-cc-pVDZ [28–30] as implemented in Psi4 [3–5]. The resulting ensemble of interaction energies is then averaged and plotted as a function of R. This same procedure is repeated for several values of R to create an approximate interaction energy curve, as shown in Supplementary Figure 4-B. As mention before, the MD trajectories can be obtained from classical or PIMD simulations, allowing to approximate the change of intermolecular interactions due to the inclusion of nuclear quantum effects.

In order to generate the results in Supplementary Figure 5 of the main text, classical MD and PIMD (with 16 beads) simulations were performed at 300K and sGDML@CCSD(T) level of theory. Then following the method described above and in Supplementary Figure 4 the interaction energy curves were generated for the three configurations of benzene pairs. Each one of the ensembles of benzene pairs considered for a given separation R was formed by 100 molecular pairs. The resulting energy curves are the defined by the expectation energy value of each ensemble. Additionally, calculations estimating the increase in noncovalent interactions were performed for the methane dimer and the methane–benzene complex as shown in Supplementary Figure 5.

SUPPLEMENTARY REFERENCES

[1] H. E. Sauceda, S. Chmiela, I. Poltavsky, K.-R. Müller, and A. Tkatchenko, "Construction of machine learned force fields with quantum chemical accuracy: Applications and chemical insights," in *Machine Learning Meets Quantum Physics*, edited by K. T. Schütt, S. Chmiela, O. A. von Lilienfeld, A. Tkatchenko, K. Tsuda, and K.-R. Müller (Springer International Publishing, 2020) pp. 277–307.

[2] S. Chmiela, A. Tkatchenko, H. E. Sauceda, I. Poltavsky, K. T. Schütt, and K.-R. Müller, Sci. Adv. **3**, e1603015 (2017).

[3] J. M. Turney, A. C. Simmonett, R. M. Parrish, E. G. Hohenstein, F. A. Evangelista, J. T. Fermann, B. J. Mintz, L. A. Burns, J. J. Wilke, M. L. Abrams, N. J. Russ, M. L. Leininger, C. L. Janssen, E. T. Seidl, W. D. Allen, H. F. Schaefer, R. A. King, E. F. Valeev, C. D. Sherrill, and T. D. Crawford, WIREs Comput. Mol. Sci. **2**, 556 (2012).

[4] R. M. Parrish, L. A. Burns, D. G. A. Smith, A. C. Simmonett, A. E. DePrince, E. G. Hohenstein, U. Bozkaya, A. Y. Sokolov, R. Di Remigio, R. M. Richard, J. F. Gonthier, A. M. James, H. R. McAlexander, A. Kumar, M. Saitow, X. Wang, B. P. Pritchard, P. Verma, H. F. Schaefer, K. Patkowski, R. A. King, E. F. Valeev, F. A. Evangelista, J. M. Turney, T. D. Crawford, and C. D. Sherrill, J. Chem. Theory Comput. **13**, 3185 (2017).

[5] D. G. A. Smith, L. A. Burns, D. A. Sirianni, D. R. Nascimento, A. Kumar, A. M. James, J. B. Schriber, T. Zhang, B. Zhang, A. S. Abbott, E. J. Berquist, M. H. Lechner, L. A. Cunha, A. G. Heide, J. M. Waldrop, T. Y. Takeshita, A. Alenaizan, D. Neuhauser, R. A. King, A. C. Simmonett, J. M. Turney, H. F. Schaefer, F. A. Evangelista, A. E. DePrince, T. D. Crawford, K. Patkowski, and C. D. Sherrill, J. Chem. Theory Comput. **14**, 3504 (2018).

[6] V. Blum, R. Gehrke, F. Hanke, P. Havu, V. Havu, X. Ren, K. Reuter, and M. Scheffler, Comput. Phys. Commun. **180**, 2175 (2009).

[7] E. D. Glendening, J. K. Badenhoop, A. E. Reed,

J. E. Carpenter, J. A. Bohmann, C. M. Morales, P. Karafiloglou, C. R. Landis, and F. Weinhold, "NBO 7.0," (2018).

[8] F. Neese, Wiley Interdiscip. Rev. Comput. Mol. Sci. **2**, 73 (2012).

[9] F. Neese, Wiley Interdiscip. Rev. Comput. Mol. Sci. **8**, e1327 (2018).

[10] H. B. Bürgi, J. D. Dunitz, and E. Shefter, Acta Crystallogr. Sect. B Struct. Crystallogr. Cryst. Chem. **30**, 1517 (1974).

[11] S. K. Singh, K. K. Mishra, N. Sharma, and A. Das, Angew. Chemie - Int. Ed. **55**, 7801 (2016).

[12] R. W. Newberry and R. T. Raines, Acc. Chem. Res. **50**, 1838 (2017).

[13] A. Choudhary, K. J. Kamer, and R. T. Raines, J. Org. Chem. **76**, 7933 (2011).

[14] M. Ceriotti, J. More, and D. E. Manolopoulos, Comput. Phys. Commun. **185**, 1019 (2014).

[15] V. Kapil, M. Rossi, O. Marsalek, R. Petraglia, Y. Litman, T. Spura, B. Cheng, A. Cuzzocrea, R. H. Meißner, D. M. Wilkins, B. A. Helfrecht, P. Juda, S. P. Bienvenue, W. Fang, J. Kessler, I. Poltavsky, S. Vandenbrande, J. Wieme, C. Corminboeuf, T. D. Kühne, D. E. Manolopoulos, T. E. Markland, J. O. Richardson, A. Tkatchenko, G. A. Tribello, V. V. Speybroeck], and M. Ceriotti, Comput. Phys. Commun. **236**, 214 (2019).

[16] S. Chmiela, H. E. Sauceda, I. Poltavsky, K.-R. Müller, and A. Tkatchenko, Comput. Phys. Commun. **240**, 38 (2019).

[17] S. Chmiela, H. E. Sauceda, K.-R. Müller, and A. Tkatchenko, Nat. Commun. **9**, 3887 (2018).

[18] K. T. Schütt, H. E. Sauceda, P.-J. Kindermans, A. Tkatchenko, and K.-R. Müller, J. Chem. Phys. **148**, 241722 (2018).

[19] I. Poltavsky, R. A. DiStasio, and A. Tkatchenko, J. Chem. Phys. **148**, 102325 (2018).

[20] A. Tkatchenko, R. A. DiStasio, R. Car, and M. Scheffler, Phys. Rev. Lett. **108**, 236402 (2012).

[21] A. Ambrosetti, A. M. Reilly, R. A. DiStasio, and A. Tkatchenko, J. Chem. Phys. **140**, 18A508 (2014).

[22] J. Hermann, R. A. DiStasio, and A. Tkatchenko, Chem. Rev. **117**, 4714 (2017).

[23] S. Grimme, A. Hansen, J. G. Brandenburg, and C. Bannwarth, Chem. Rev. **116**, 5105 (2016).

[24] T. Liljefors and N. L. Allinger, J. Comput. Chem. **6**, 478 (1985).

[25] T. Kundu, B. Pradhan, and B. P. Singh, J. Chem. Sci. **114**, 623 (2002).

[26] P. George, C. W. Bock, J. J. Stezowski, T. Hildenbrand, and J. P. Glusker, J. Phys. Chem. **92**, 5656 (1988).

[27] P. J. Breen, J. A. Warren, E. R. Bernstein, and J. I. Seeman, J. Chem. Phys. **87**, 1917 (1987).

[28] B. Jeziorski, R. Moszynski, and K. Szalewicz, Chem. Rev. **94**, 1887 (1994).

[29] R. M. Parrish, E. G. Hohenstein, and C. D. Sherrill, J. Chem. Phys. **139**, 174102 (2013).

[30] T. M. Parker, L. A. Burns, R. M. Parrish, A. G. Ryno, and C. D. Sherrill, J. Chem. Phys. **140**, 094106 (2014).
